# Accurate recombination estimation from pooled genotyping and sequencing: a case study on barley

**DOI:** 10.1186/s12864-022-08701-7

**Published:** 2022-06-25

**Authors:** Michael Schneider, Federico Casale, Benjamin Stich

**Affiliations:** 1 Institute of Quantitative Genetics and Genomics of Plants, Heinrich Heine University, 40225 Düsseldorf, Germany; 2 Max Planck Institute for Plant Breeding Research, 50829 Köln, Germany; 3 Cluster of Excellence on Plant Sciences, From Complex Traits Towards Synthetic Modules, Universitätsstraße 1, 40225 Düsseldorf, Germany

**Keywords:** Recombination rate, Pool sequencing, Population genetics, Genetic map, Breeding value estimation

## Abstract

Sexual reproduction involves meiotic recombination and the creation of crossing over between homologous chromosomes, which leads to new allele combinations. We present a new approach that uses the allele frequency differences and the physical distance of neighboring polymorphisms to estimate the recombination rate from pool genotyping or sequencing. This allows a considerable cost reduction compared to conventional mapping based on genotyping or sequencing data of single individuals. We evaluated the approach based on computer simulations at various genotyping depths and population sizes as well as applied it to experimental data of 45 barley populations, comprising 4182 RIL. High correlations between the recombination rates from this new pool genetic mapping approach and conventional mapping in simulated and experimental barley populations were observed. The proposed method therefore provides a reliable genetic map position and recombination rate estimation in defined genomic windows.

**Supplementary Information:**

The online version contains supplementary material available at 10.1186/s12864-022-08701-7.

## Introduction

Sexual reproduction involves meiotic recombination and the creation of crossing over between homologous chromosomes, which leads to new allele combinations [1]. The resulting phenotypic diversity is the basis of evolution and human selection [2]. Meiotic recombination is therefore essential in various research fields such as medicine, animal and plant breeding, conservational and evolutionary genomics [2–8]. Especially in breeding, the response to selection is strongly associated with the recombination rate. Therefore, increased recombination can enhance breeding and selection efficiency [9]. Besides, a high recombination rate could foster the dissociating of phenotypic and genetic variation [10] and affect reproductive barriers.

The exchange mentioned above between homologous chromosomes was first reported by T.H. Morgan, who identified novel allele combinations after crossing two *Drosophila melanogaster* strains [11–13]. Since then, incredible progress has been made in uncovering the molecular mechanisms of meiotic recombination [14, 15]. Furthermore, interest increases in understanding the effect of environmental factors on the recombination rate (RR) or the inter- and intraspecies variation of RR (e.g. [15–18]).

Detecting differences in RR among environmental conditions, genetic backgrounds, or species requires the genotypic characterization of a representative number of genotypes of each treatment. The most frequently applied genotyping approach in this context is using SNP arrays. However, the main limitation of such approaches is that the costs increase linearly with the number of evaluated genotypes. Furthermore, the number of loci typically genotyped with SNP arrays is limited to a few thousand variants [19–23]. This limits the resolution of the resulting genetic map, which hinders, e.g., studies on populations with a long history of natural or artificial selection [24]. Sequencing strategies like genotyping by sequencing [25, 26], exome capture [27, 28], whole-genome resequencing [29, 30], or RNA sequencing [31, 32] are useful to increase the genome-wide variant density and coverage. However, such approaches applied to individual genotypes have the same limitations as mentioned above for SNP array genotyping – the costs increase linearly with the number of studied genotypes.

The progress of sequencing techniques allowed the estimation of recombination events from linked read gamete sequencing [33]. Although this approach revealed promising results, the high experimental effort and associated costs might prevent its implementation in extensive recombination screening studies.

Our study proposes an alternative approach to overcome the burden of either high costs, low variant densities, or low genotype count. The proposed method allows the estimation of the RR from pooled genotype samples. In this situation, any user-defined quantity of genotypes can be pooled without increasing the monetary costs of genotyping or sequencing. Our approach uses the allele frequency differences and the physical distance of neighboring polymorphisms to estimate the RR, an idea initially proposed for situations with a linked locus under selection that causes a fitness differential [34].

The objectives of our study were

i. to assess the accuracy of estimated genetic maps and RR from pool genotyping based on computer simulations,
ii. describe a best practice guideline for accurate RR extraction from pool genotyping and sequencing, and
iii. apply the RR estimation on experimental populations of barley

## Results

### Raw pool genetic map (PGM) calculation from simulated populations

We simulated 1260 F_2_ populations with various genotyping depths and population sizes. The simulations were performed based on a consensus genetic map calculated for 4182 recombinant inbred lines from 45 barley HvDRR populations [18].

The genome-wide SNP allele frequency observed in the simulated populations deviated from the expected 0.5 (Supplementary Figure 1). The average deviation was highest in small populations (50 genotypes – 0.04, standard deviation = 0.03). It decreased exponentially to a genome-wide average of 0.003 (sd = 0.002) for the populations consisting of 10,000 genotypes.

**Fig. 1:**
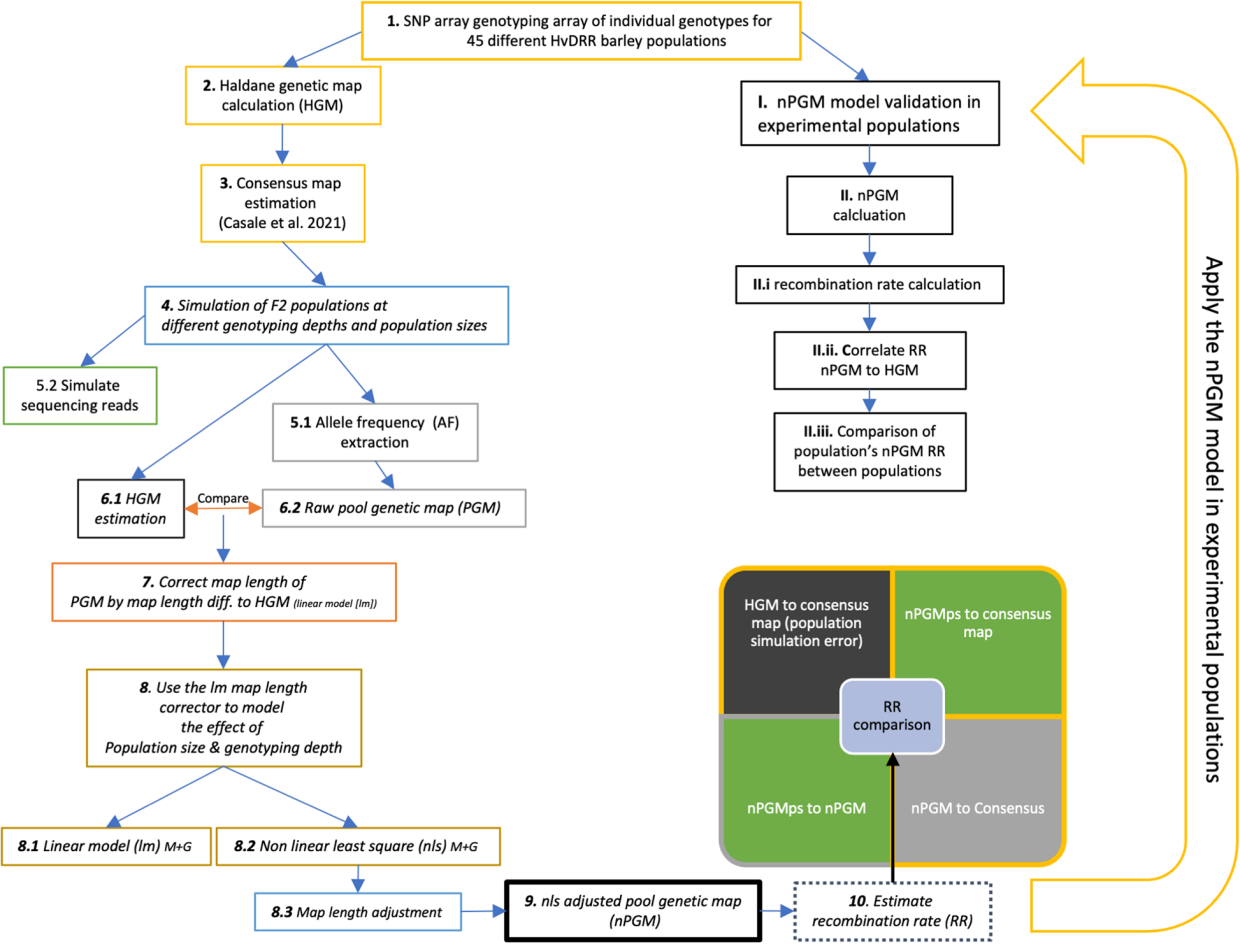
Workflow of performed analysis and steps to retrieve a recombination rate (RR) estimate from pooled samples. Box outline color and fill color refer to the same group of performed calculations

**Fig. 2:**
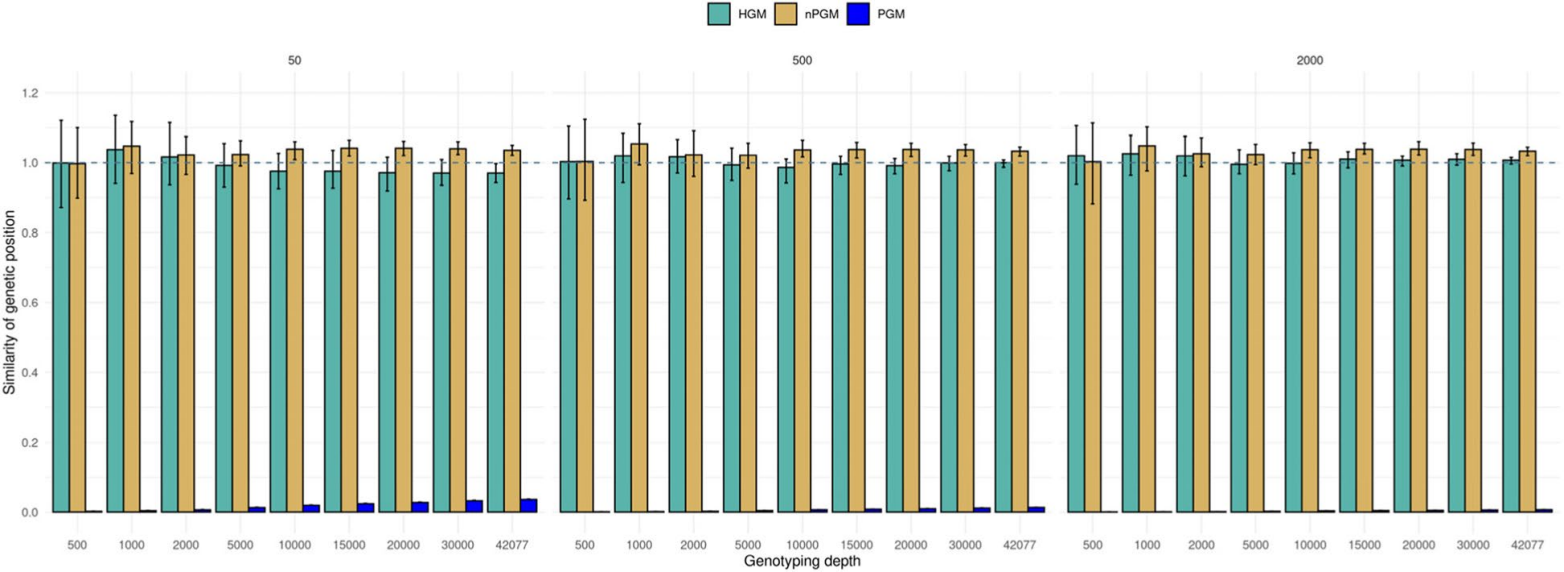
Effect of genotyping depth on the accuracy of the genetic position in PGM estimations. A ratio of PGM (blue), nPGM (golden), and HGM (turquoise) genetic map position compared to the true map position, namely the consensus genetic map, was calculated for each marker. A level of 1 indicates a perfect position match (dashed line). The error bars indicate the standard deviation over the replicates. Sub figures present the impact of different population sizes. HGM was calculated from individual genotyping, while (n)PGM was calculated from pooled genotyping. nPGM was PGM adjusted by least squares, PGM is unadjusted

**Fig. 3:**
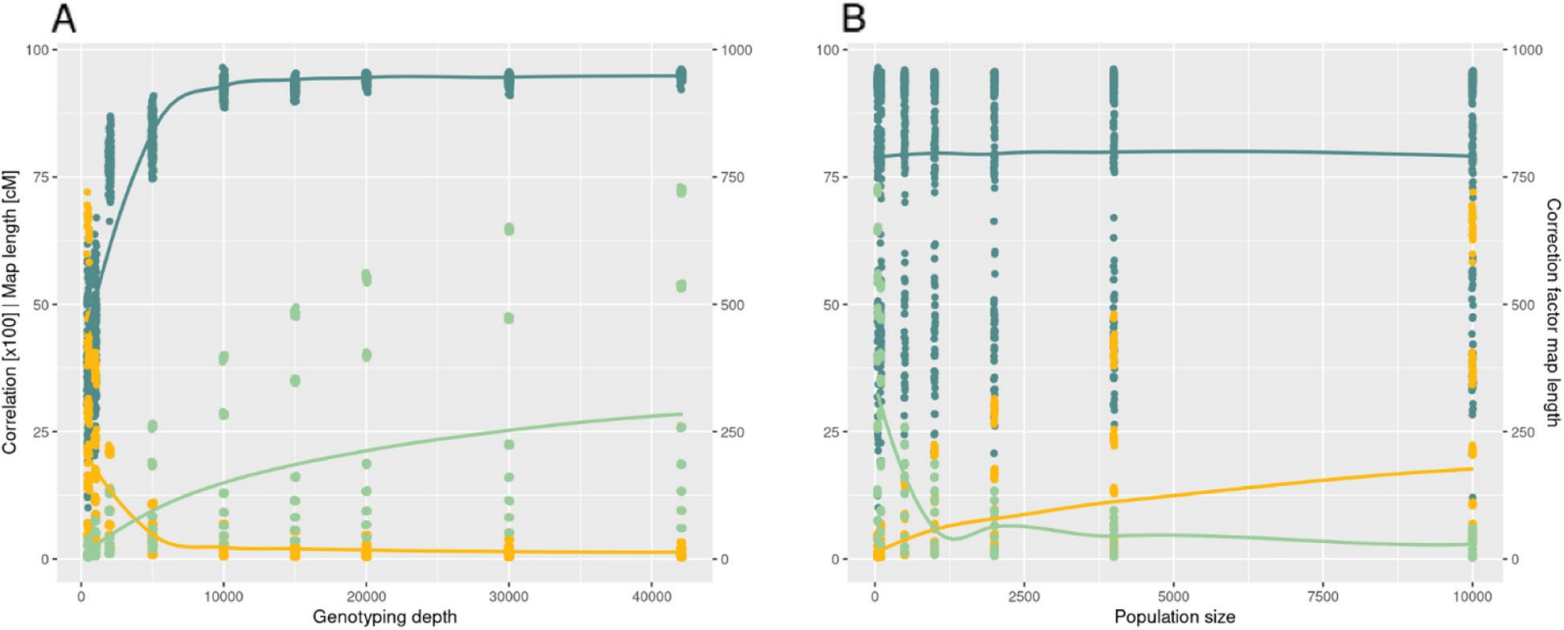
Correlation coefficient of the recombination rate between PGM and consensus map (petrol), map length (light green), and the correction factor (yellow) for the PGM (Step 7 & 8 from Fig. 1) for varying genotyping depths (**A**) and the population sizes (**B**). Lines indicate the smooth loess curve

Based on the allele frequency deviation of pairs of physically neighboring SNPs and their physical distance, we estimated the raw pool genetic map (PGM) and calculated the PGM recombination rate (RR_PGM_) for 50 MB windows across the genome (Fig. 1). The average correlation coefficient of the RR derived from the consensus genetic map (RR_consensus_) and the RR_PGM_ was r = 0.894 across all genotyping depths. The lowest correlation was observed when only 500 markers were used for genotyping the population (r = 0.819, Table 1). Generally, a continuous increase in the correlation between RR_PGM_ to RR_consensus_ was observed with increasing genotyping depth, where a maximum Pearson correlation of 0.994 was observed for a genotyping depth of 42,077 (Table 1). Despite the above described high correlation coefficients between RR_consensus_ and RR_PGM_, we observed that the average PGM to consensus genetic map position ratio was 0.0093, indicating a significant underestimation of the overall PGM length and RR_PGM_ (Fig. 2). Additionally, the PGM’s standard deviation across all samples was 0.01–1,1 times the average genetic map length ratio. Therefore, we investigated the effect of the genotyping depth and the population size on the length of the PGM and the accuracy of the RR_PGM_ estimation. We observed a shorter PGM in those simulated samples with a low genotyping depth and an almost linear increase in map length with increasing genotyping depth (Fig. 3A). Analogously, the population size influenced the overall extent of the genetic map length and RR_PGM_. We noticed a decrease in genetic map length with increasing population size (Fig. 3B). In contrast to the genotyping depth, no effect of the population size was observed on the correlation between RR_PGM_ to RR_consensus_.

**Table 1:** Evaluation of the precision and accuracy of the adjusted pool genetic map derived recombination rate (RR_nPGM_) in comparison to RR_consensus_ on varying levels of the genotyping depth

Genotyping depth	RMSE		Pearson correlation	
	*average*	*SD*	*average*	*SD*
*500*	0.8597	0.00027	0.387	0.1037
*1000*	0.4596	0.00029	0.446	0.0939
*2000*	0.2526	0.00028	0.787	0.0357
*5000*	0.1168	0.00026	0.835	0.0406
*10,000*	0.0673	0.00023	0.925	0.0150
*15,000*	0.0510	0.00020	0.928	0.0120
*20,000*	0.0405	0.00018	0.941	0.0080
*30,000*	0.0312	0.00015	0.941	0.0080
*42,077*	0.0250	0.00014	0.950	0.0070

*SD* Standard deviation, *RMSE* Root mean square error

In order to obtain a PGM with a length as close as possible to that of the consensus map, correction approaches were investigated. We evaluated the use of two models that included effects for the genotyping depth and population size: a linear and a non-linear model. While the linear model revealed a log-likelihood of 10,332 and an AIC of 20,672, the non-linear model resulted in a log-likelihood and AIC values 30 and 26% lower than that of the linear model, respectively. This was accompanied by a Pearson correlation between the RR_PGM_ and the RR_consensus_ of 0.635 for the linear model and 0.998 for the non-linear model (Supplementary Figure 2). Therefore, we used the latter to correct the SNP’s genetic position on the PGM to a non-linear adjusted pool genetic map position (nPGM).

### Non-linear adjusted pool genetic map and derived recombination rate

After utilizing the above described non-linear adjustment, the nPGM estimated genetic map positions deviated marginally from the consensus map (Fig. 2). Across all tested samples, each marker’s average nPGM to consensus map position ratio was 1.03, which was very close to the ratio of the Haldane genetic map (HGM) to consensus map (ratio = 1.00). HGM is the genetic map recalculated from simulated samples by the Haldane mapping approach. In addition, nPGM resulted in a lower relative standard deviation across all population sizes and genotyping depth than PGM (sd = 0.014).

**Fig. 4:**
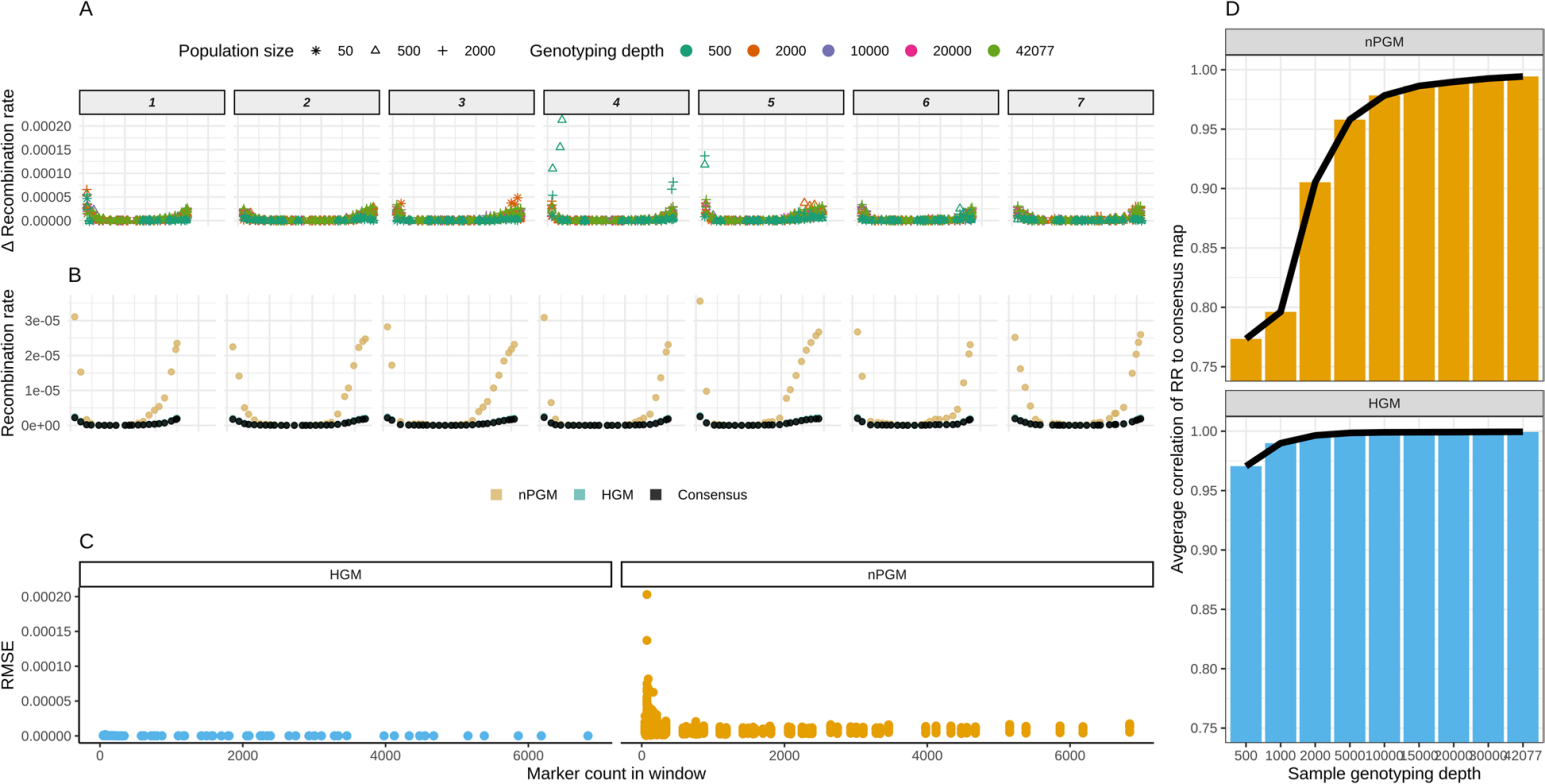
Assessment of recombination rate; **A** The recombination rate’s RMSE between HGM and the consensus map and nPGM and the consensus map regarding marker count in windows. **B** Average correlation coefficient of the nPGM and HGM recombination rate compared to the consensus map in 50 MB windows regarding variant genotyping depths. **C** Deviation of the cM/MB recombination rate, assessed by nPGM, from the consensus map. Value depends on the physical position (x-axis), the genotyping density (color), and the population (shape). The genome is divided by chromosomes (gray blocks on top) **D** Observed recombination fraction (square rooted for better visualization purpose; cM/MB) for nPGM (gold), Haldane genetic map (turquoise), and the consensus map (gray). 42.077 variants and a genotyping depth of 2000 were used to simulate the population. For this set, the recombination rate in 50 MB windows was calculated. **E** Estimated genetic map position (y-axis) in relation to the physical position (x-axis) for the same sample as illustrated in D. The genetic map position is the result of aggregating the single marker recombination rate along the chromosomes. Color scheme identical to subfigure D

To compare the RR_HGM_ and RR_nPGM_ to the RR_consensus_, we calculated the RR in genomic windows of 50 MB for all replicates of the simulated samples with a population size of 50, 500, and 2000 at all genotyping depths. We observed significant RR correlations between HGM and the consensus map across all tested SNP and genotype levels (correlation test *p* < 2 × 10^− 16^), with an overall Pearson correlation of 0.973. The correlation increased to 0.999 when excluding those samples with a genotyping depth below 10,000 markers (Fig. 4B). Similarly, we observed an average Pearson correlation of 0.913 between the RR_nPGM_ and RR_consensus_ for those samples with a genotyping depth ≥ 10,000 (Fig. 4B). Furthermore, we noted a significant effect of the number of markers in the 50 MB windows on the correlation coefficient and the RMSE in the RR_HGM_ and RR_nPGM_ estimations (*p* < 0.0001, Fig. 4A). The RMSE of nPGM decreased by a factor of four in genomic windows with more than 1000 markers compared to windows with less than 100 markers. In contrast, the RMSE decrease was only 1.17 times for HGM for the same comparison. Analogously, samples characterized by a low genotyping depth resulted in a lower SNP density in genomic windows and, thus, resulted in an increased deviation of RR_nPGM_ (Fig. 4C). In the last step, we compared the absolute recombination rates on the chromosomal scale among the three approaches (Fig. 4D). The RR was highly similar between HGM and the consensus map throughout the entire genome (r > 0.98). In analogy, we observed high similarities in the pericentromeric regions when comparing nPGM and the consensus map. Nevertheless, the non-pericentromeric regions revealed a more pronounced deviation of RR – especially on the long chromosomal arms. However, the Pearson correlation coefficient between the RR_nPGM_ and RR_consensus_ remained high with r_non-pericentromeric_ = 0.782 vs. r_pericentromeric_ = 0.915 for samples with a genotyping depth ≥ 10,000 across all chromosomes and replicates. However, the deviations of the RR, estimated from nPGM compared to that of the consensus map, only minorly altered the marker’s genetic map position (Fig. 4E).

### nPGM estimation in experimental populations

In addition to simulations, we were interested in using the nPGM approach in experimental populations. Therefore, we applied the nPGM strategy to a set of 45 segregating spring barley populations [33], characterized by an average of 87 recombinant inbred lines (RIL) per population and an average number of 1639 polymorphic SNPs. Pooled genotyping information for all populations was derived from the available genotyping data of individual RIL, and the nPGM and nPGM-derived RR were calculated and compared to HGM-derived values.

**Fig. 5:**
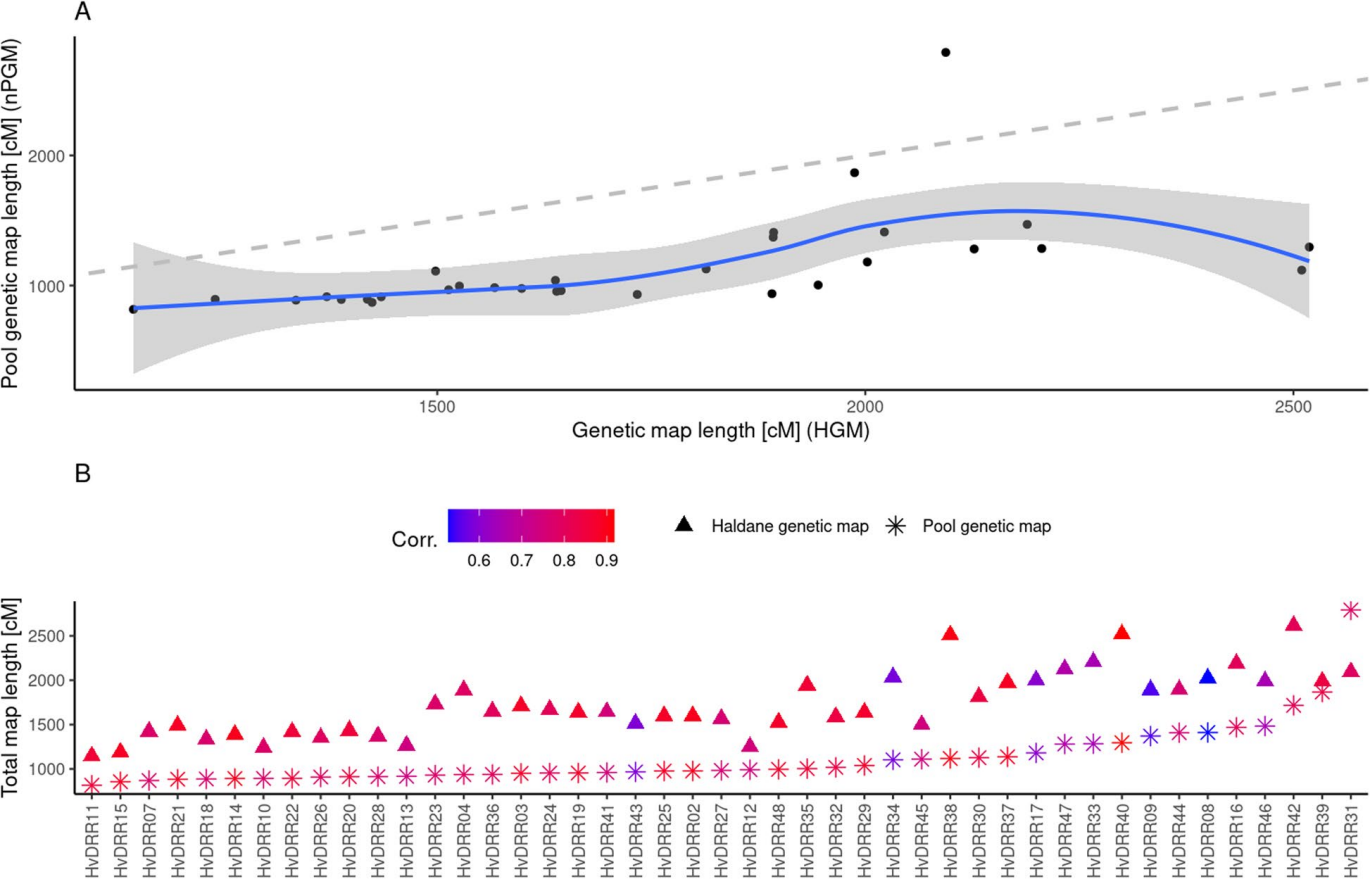
Comparison of HGM to nPGM for 45 HvDRR populations. **A** Compare map length between HGM (x-axis) and nPGM (y-axis). The gray dashed line indicates expectation, while the blue line indicates a loess curve over the black data points. The gray area illustrates the 95% confidence interval. **B** The total map length for each population across all seven chromosomes. The mapping algorithm is differentiated by shape. The color denotes the genome-wide recombination rate correlation between the HGM and nPGM for the particular population, based on the recombination rate of 50 MB windows

Across all 45 populations, an average Pearson correlation of 0.829 was observed between the RR_HGM_ and the RR_nPGM_ in 50 MB windows (95% Confidence interval r = 0.37:0.95, correlation test *p* < 5 × 10^− 10^; Fig. 5B).

We observed a similar range of map length across all populations for the nPGM approach (90% confidence interval 873:1670 cM) compared to the HGM (90% confidence interval 1242:2449 cM) (Fig. 5A). Nevertheless, the overall map length was, on average, across all populations, 635 cM longer in HGM than PGM (Fig. 5B). Spearman rank sum correlation between HGM and nPGM revealed a high correlation of 0.83, whereas the Pearson correlation was 0.61.

To evaluate whether the accuracy of the nPGM approach is sufficient to detect differences among the RR_HGM_ and RR_nPGM_, we used the genome-wide RR_nPGM_ to estimate a general recombination effect (GRE) for each of the 23 parental inbreds, as was proposed by [18]. This step revealed considerable variations in the GRE among parental inbreds, indicating that some inbreds result in a higher RR_nPGM_ in their progenies than others (Suppl. Figure 4). The direct comparison of the GRE, calculated from RR_nPGM,_ with the RR_HGM_ GRE from Casale et al. (2021), revealed a rank-sum correlation of 0.877, indicating high similarities (Pearson correlation = 0.803). In the group of the ten genotypes with the highest GRE, nine matched between nPGM and HGM. Similarly, eight of ten genotypes with the lowest GRE were identical between nPGM and HGM.

### Effect of sequencing bias on nPGM accuracy

Next, we evaluated the genetic map estimation from pooled sequencing data using simulated reads for 42,077 and 10,000 SNPs and three sequencing depths. Limitations of the simulation software did not allow reliable simulations with more than 100 genotypes; thus, we evaluated population sizes of 50 and 100. After simulation, variant calling, and allele frequency estimation, the genetic map of the simulated pool sequencing was calculated (nPGM_ps_), and the corresponding RR_nPGMps_ was assessed.

**Fig. 6:**
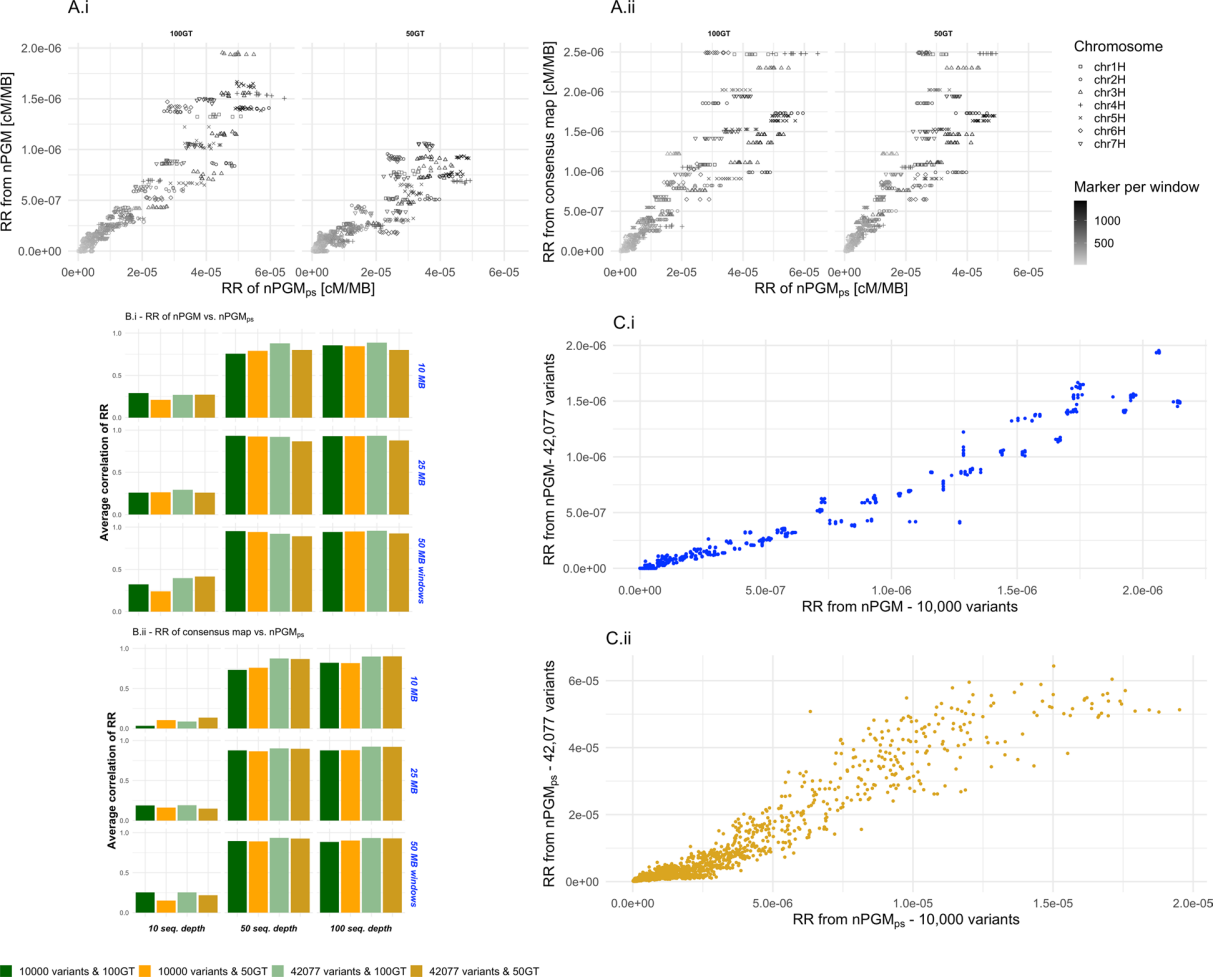
Pool sequencing (nPGM_ps_) to estimate recombination rates (RR) in genomic windows of 50 MB. **A.i** Correlation plot of the RR from pooled genotyping (RR_nPGM_, y-axis) compared to the RR from simulated pool sequencing (RR_nPGMps_, x-axis) at 100 reads coverage for 42,077 variant loci at population sizes of 50 and 100. **A.ii** Similar to A.i, but comparing the RR_nPGMps_ to RR_consensus_. The number of variants in the genomic windows is illustrated by color, while the chromosomes are differentiated by shape. A larger scale of cM/MB was observed for nPGM_ps_ than nPGM and the consensus map. **B** The average RR across 20 simulated replicated samples at given sequencing depth (x-axis) and the associated genomic window (y-axis). The samples are differentiated by color. **B.i** Correlation coefficient between RR_nPGM_ and RR_nPGMps_. **B.ii** Correlation coefficient between RR_nPGMps_ and RR_consensus_. **C** Illustrating the effect of different genotyping depths on the correlation coefficient of RR on genotyping (**C.i**) and pool sequencing level (**C.ii**). **C.i** Comparing RR_nPGM_ between samples with 42,077 (y-axis) and 10,000 (x-axis) variants genotyping depths; **C.ii** Similar to C.i on RR_nPGMps_

While the population size and genotyping depth did not significantly (P_popSize_ = 0.21; P_genotypingDepth_ = 0.56) affect the RR_nPGMps_ estimation accuracy, the sequencing depth and the genomic window size significantly (P_seqDepth_ < 0.0001; P_genWindow_ < 0.001) impacted the accuracy. When RR_nPGM_ and RR_nPGMps_ were compared based on a shallow sequencing depth of 10 reads per locus, we observed a low Pearson correlation of 0.26 between them (Fig. 6B.i). However, the correlation coefficient increased to 0.88 and 0.9 for sequencing depth of 50 and 100 reads/locus (Fig. 6A.i & B.i).

The correlation coefficients between RR_nPGMps_ and RR_nPGM_, estimated in genomic windows of 10 MB, were about 90% lower than that observed for 50 MB windows. This was caused by the low SNP density in the 10 MB windows. Generally, the highest correlation was observed for a read depth of 100 in 50 MB windows (r = 0.94, Suppl. Figure 5).

Similar patterns of Pearson correlation coefficients were observed when comparing RR_nPGMps_ and RR_consensus_. The highest correlation was detected for genomic windows of 50 MB and a sequencing depth of 100 (r = 0.912, Fig. 6A.ii & B.ii). In contrast to the comparison between RR_nPGM_ and RR_nPGMps_, where the correlation was higher for 10,000 than 42,077 variant loci, a higher correlation coefficient (r = 0.918) was observed for the scenario with 42,077 variant loci compared to that with 10,000 variant loci (r = 0.863) when considering the comparison between RR_nPGMps_ and RR_consensus_.

The assessment of the absolute levels of the RR_nPGMps_ based on the different population sizes (Fig. 6C) revealed no influence of this parameter (Fig. 6C.i, 1:1 ratio to RR_nPGM_). However, the genotyping depth strongly impacted the absolute value of the RR_nPGMps_ (Fig. 6C.ii, 4:1 ratio to RR_nPGM_).

Similarly, we observed a mean overestimation of RR_nPGMps_ to RR_PGM_ of 16.7 times (Fig. 6A, axis scales). This observation indicates that the sequencing procedure likely adds extra allele frequency deviations.

## Discussion

### Need for cheap RR estimation

The accurate and cost-efficient RR estimation of populations, lines, species, or genetic material that experienced different environmental cues is a technique many research fields could benefit from [35–39]. Commonly used approaches to estimate recombination rates require both the haplotype and allele frequency [40]. This information is typically derived from genotyping or sequencing of single individuals. However, crossing-overs per chromosome and meiosis are typically limited to one to four [28]. Therefore, many individuals must be genotyped to obtain accurate recombination rate estimations [41].

### Concept of pool-based RR estimation and its evaluation using computer simulations

This study describes an approach for RR estimation that does not require genotyping or sequencing of single individuals – without considerable sacrificing accuracy. The method relies on two sources of information: (i) the allele frequency at each polymorphic locus and (ii) the physical position of these loci. We followed the idea that the allele frequency difference of two neighboring polymorph loci indicates a crossing-over [34]. Thus, our approach does not require collecting haplotype information by genotyping single individuals.

The required allele frequency variations across the genome are caused by the combination of migration, selection, drift, or gene flow [42]. However, even unintended selection or drift can result in traceable allele frequency deviations in populations (Suppl. Figure 1).

The extent of cross-overs can be quantified based on this concept, but the effective RR cannot be derived from allele frequency variations alone. For example, recombination between two loci with a distance of one Mb is much less likely than between two loci separated by 10 Mb. Suppose the allele frequency variation in both situations is identical. In this case, the recombination likelihood in the small interval is much lower; therefore, the local RR must be higher than that of the big interval. Accordingly, we scaled the allele frequency deviation by the log_10_ of the physical distance of the considered loci to calculate parameters related to the local RR (Eq. 1).

The first objective of our study was to propose an accurate and reliable method for genetic map position and RR estimation in defined genomic windows. For a genotyping depth above 10,000 markers, we observed a high correlation coefficient (r > 0.9) between RR_PGM_ and RR_consensus_ (Fig. 3). Nevertheless, the actual PGM map length and the extent of RR_PGM_ were (i) underestimated and (ii) depended on the genotyping depth and population size (Figs. 2 & 3). Exemplarily, the average map length in the simulated samples with only 500 SNPs was 20 times shorter than the map length of samples with 42.077 SNPs (Fig. 2). This can be explained thereby that with increasing genotyping depth, undetected recombination in maps with fewer loci will be observed, which increases the recombination rate. From this observation, we concluded that it is crucial to integrate the number of polymorph loci in the RR_PGM_ estimation. Especially when only a few polymorph loci are available, the variation in genotyping depth between two populations might affect the comparison. In addition, we also observed an effect of the population size (Fig. 3) on RR_PGM_. Fewer genotypes resulted in a higher deviation of the actual allele frequency, which resulted in a higher RR_PGM_ estimate than expected. In analogy to the genotyping depth, this might not be relevant in weakly unbalanced experimental designs, but an adjustment might prevent the overestimation of RR_PGM_.

Therefore, a linear and a non-linear model were examined to adjust the extent of RR_PGM_ by considering the genotyping depth and the population size. The non-linear least square model performed superior to the linear model (Suppl. Figure 2). The final model implemented in the further comparisons was:

$$n=7958.92\ast {e}^{-0.5401\ast {\mathit{\log}}_2\sum SNPs}\ast {e}^{0.3491\ast {\mathit{\log}}_2\sum Genotypes}+\frac{691.0495}{\sqrt{\sum SNPs}}$$

Using this model’s result, multiplied with the outcome of *eq.* 1, provides an unbiased estimation of the recombination rate. This adjustment of PGM to nPGM resulted in genetic maps having the same map extension as HGM (Fig. 2), regardless of the genotyping depth (Σ SNPs) or population size (Σ Genotypes). Furthermore, we could show that the correlation coefficient between RR_nPGM_ and RR_consensus_ was only slightly lower than the correlation coefficient between RR_consensus_ and RR_HGM_ (Fig. 4A & C). Especially when the genotyping depth was high, the correlation coefficients were almost identical. An even higher correlation coefficient between RR_nPGM_ and RR_HGM_ was observed than between RR_nPGM_ and RR_consensus_. This can be explained thereby that the simulation of populations introduced a measurable simulation error. These observations indicated that RR estimation from pooled samples is possible with high accuracy at dramatically reduced costs.

In addition to the correlation of the recombination rates, we evaluated the accuracy of the RR_nPGM_ estimation on a genome-wide scale. This analysis indicated an overestimation of the RR_nPGM_ in non-pericentromeric regions of the genome (Fig. 4D). This deviation is presumably caused by the different variant distribution of the SNP array in the non-pericentromeric compared to the pericentromeric region. Therefore, the observed overestimation of RR_non-pericentromeric_ regions is only problematic if the RR is compared between different genotyping approaches.

### Pool-based RR estimation in experimental populations

The 45 HvDRR populations were characterized by varying genotyping depth (deviation of lowest to highest – 5.79x) and population sizes (variation of smallest to largest– 3.76x). Therefore we applied the nPGM approach to adjust for genotyping depth and population size. Although we used the nPGM model described above, we observed a map length that was, across all populations, about 33% lower compared to the HGM reported by Casale et al. (2021) (Fig. 5). This observation can be explained thereby that the experimental populations are RIL populations, while the model underlying the nPGM approach was established based on simulated F2 populations. The total number of recombination events accumulated in the gametes of a RIL, after endless selfing generations, was about twice the number of such events in an F2 population [43]. Therefore, if the absolute value of the map positions is of interest, then the model underlying nPGM approach needs to be derived de novo for the population type under consideration. However, in analogy to the results of the simulations, the map length variations did not affect the correlation of the RR_HGM_ to RR_nPGM_, which was r > 0.8 for 23 of 45 populations (r_Spearman_ > 0.6 for 41 populations, Fig. 5B). We explored potential reasons for these deviations and observed that the populations with a correlation of the RR_HGM_ to RR_nPGM_ < 0.6 were characterized by a median inter-marker distance that was about 40% lower than that of the other populations (Suppl. Figure 6). We tested this effect for statistical significance in a linear model and retained a significant effect of the genome-wide median inter marker distance and standard deviation on the Pearson correlation of RR_nPGM_ to RR_HGM_ (p_Median_ < 0.003; p_Sd_ < 0.002). Similarly, we observed the same effect on the Spearman correlation (p_Median_ = 0.001; p_Sd_ = 0.0015). Contrary, no genotyping depth or population size effect was observed (p_GD_ = 0.34; p_PS_ = 0.33). We conclude from this observation that a skewed distribution of genomic marker distances can significantly affect the RR_nPGM_ estimation. One possibility to overcome this problem is to sample the loci such that all loci with a distance below 10,000 bp are omitted for further progression with the nPGM approach. However, this requires further research.

Subsequently, we were interested in comparing the general recombination estimate (GRE) derived from the nPGM approach with that from HGM. This parameter summarizes the RR of a parental genotype in combination with several parental genotypes and is highly relevant for breeders of all crops, exemplarily in introgression breeding [8]. Compared to the HGM-based GRE of Casale et al. (2021), the GRE calculated from nPGM resulted in almost the same ranking of the involved 23 parental inbreds (Suppl. Figure 4). Deviations in the ranking between nPGM and HGM-derived GRE might be due to discrepancies between the genetic and physical order of the underlying marker (Suppl. Figure 3), which either can be artifacts from the HGM approach or are structural variants in the genomes of some of the parental inbreds.

These observations together illustrated the validity and accuracy of RR estimates from nPGM also in experimental populations.

### Pool-based RR estimation by sequencing

For the above-described results, we derived pool genotyping data from genotyping information of individuals as a starting point for evaluating our approach. This procedure results in the upper limit of the accuracy as it neglects the variation in allele frequency that is caused by its estimation in a pool. One possibility would be to consider this aspect in our simulations of genotyping with an SNP array. However, with today’s sequencing costs [43], applying our method to datasets created from the sequencing of pooled samples is even more economically attractive. Therefore, we estimated the accuracy of recombination rate estimation from simulated pool sequencing samples. The correlation between RR_consensus_ and RR_nPGMps_ was, at a coverage of 10 reads per locus, at a rather low level of about 0.3 (Fig. 6B.ii). However, we observed that increasing the read dept. from 10 to 50 reads per locus reduced the median variation of simulated pool sequencing compared to the RR from pool genotyping by 40% (Supple. Figure 5).

Similarly, RR_consensus_ and RR_nPGMps_ correlation increased to 0.93 in 50 MB genomic windows at 50x coverage. A further increase of the read coverage to 100 did not result in similarly high additional precision, indicating that saturation was reached. The second aspect that was studied, in addition to the sequencing depth, was the size of the genomic window for which the RR was estimated. At a sequencing depth of 100 reads, the median error was reduced by 50% when comparing the 10 to 25 MB genomic windows (RMSE 10 MB = 0.0004; 50 MB = 0.0002). The error was only further reduced by 2% comparing the RMSE of 25 and 50 MB windows (Supple. Figure 5). The choice of a reasonable window size for summarizing the RR is impacted by the number of variants present in a window. In our simulations, we assumed conservatively 10,000 and 42,077 genome-wide variant loci. However, a considerably higher number of polymorphic loci in most species will be identified when sequencing strategies are applied. For barley, e.g., more than 57 Mio. SNPs were collected in a variant database [44], indicating that more than 1350x variants than those used in our study are already known. Therefore, we expect that the window size can be considerably decreased down to less than 1 MB in future experimental studies. This in turn allows to increase the resolution.

Besides the high correlations of RR_nPGMps_ to RR_nPGM_, we noticed a significant overestimation of RR_nPGMps_ compared to RR_nPGM_ (16.7 times higher, Fig. 6A). This observation might be due to the additional allele frequency variation between adjacent loci caused by sequencing errors. However, the above-described overestimation only matters when comparing the RR among different methods, like RR_HGM_ to RR_nPGMps_.

Furthermore, we also observed a variation in the scale of RR between the genotyping depth levels. The extent of RR_nPGMps_ increased with the genotyping depth (Fig. 6C.ii). Higher genotyping depth might be associated with a smaller inter polymorphism distance and, therefore, might lead to a further increased overestimation of the RR_nPGMps_. To generate a comparison on the same scale, a simple linear model correction for the RR_nPGMps_ might be suitable to compare it to other approaches’ derived RR. Apart from this overestimation of the RR, the RR_nPGMps_, RR_nPGM,_ and RR_consensus_ indicated high similarities in the genomic window-base recombination estimation (Fig. 6B).

### Comparison of the nPGM approach to other approaches of RR estimation

Generally, the observed accuracy of our approach of estimating the RR in pooled samples might overcome issues of related approaches, like high costs, and allow a high throughput screening for GRE.

Other attempts to solve the dilemma of high costs have been proposed earlier. For example, [45] proposed an ultra-low individual sequencing strategy, followed by an imputation step to recover none sequenced regions in the library. Nevertheless, the imputation might also introduce errors in the recombination estimation, making accurate recombination estimation challenging.

Other approaches minimize the number of test samples by implementing Markov Coalescent models or machine learning strategies trained in different subsamples or even species [46, 47]. Few single genotypes need to be sequenced in these approaches to estimate the genetic map to retain haplotype information in the sample. This is based on applying genetic maps from related species might be a useful approach to estimate the RR, especially when few samples or no reference genome are available or costs should be reduced. In situations where no reference genome is available, our nPGM_ps_ method cannot be performed and is inferior to these methods. Nevertheless, the RR might differ from one species to another [48], and our proposed nPGM_ps_ approach allows differentiating populations of the same species with a much higher resolution.

Sun et al. (2019) showed that the unexpected breaks in linked read sequencing of F_1_ plants’ pollen could denote recombination events. While this approach is complex and costly to perform, generating a pooled sample with equal tissue contribution of each genotype from leaves or seeds underlying our method is technically easy. Furthermore, our method allows genotyping of undefined population sizes without cost inflations. The nPGM_ps_ method does not demand more than 10 to 100 reads coverage per locus, while the pool-linked read sequencing of haploid cells requires ultra-high sequencing depth across all the pollen. Furthermore, pool sequencing prices can be further decreased when the sequencing depth is reduced [34, 49]. The only disadvantage of our method is that it is not based on the F1 generation as the approach of Sun et al. (2019) but requires the establishment of at least the F2 generation. However, that is possible for most species without big space limitations and is more than balanced by the considerably lower costs.

### Implementation of the (n)PGM_ps_ approach in other genetic materials or species

In order to generate a genome-wide genetic map for a species of interest using the PGM approach, the following prerequisites have to be fulfilled. First, a reference sequence must be available to align short reads to annotated positions. Second, a pool sequencing strategy has to be chosen that ideally allows to remove duplicated reads (unlike restriction-site based genotyping by sequencing) and is unbiased regarding the expression level (like RNAseq). This is because such sequencing procedures can bias the accurate allele frequency estimation and therefore are less suitable for pool sequencing [50] and RR_nPGMps_ estimation. Consequently, we propose whole-genome sequencing as the most convenient method to generate high-confidence allele frequency estimates (Table 2 – 1.1-1.3). Furthermore, a sequencing depth of approximately 100x or higher will result in sufficiently accurate allele frequency estimations. Nevertheless, a 100x coverage is associated with high monetary costs, especially for crop species with large genomes, so one might want to sequence a pool on a lower coverage level (exemplarily 10x, Table 2 – 2.1-2.7). SNP allele frequency aggregation to a haplotype (window) frequency is suitable for increasing precision in such cases. The haplotype creation can either be based on defined genomic windows [49] or on genomic features, like genes [51]. However, it must be pointed out that such haplotype aggregations or generally lower counts of detected variant loci (like GBS) will reduce the RR resolution (cf. Table 2 – 1.4).

**Table 2:**
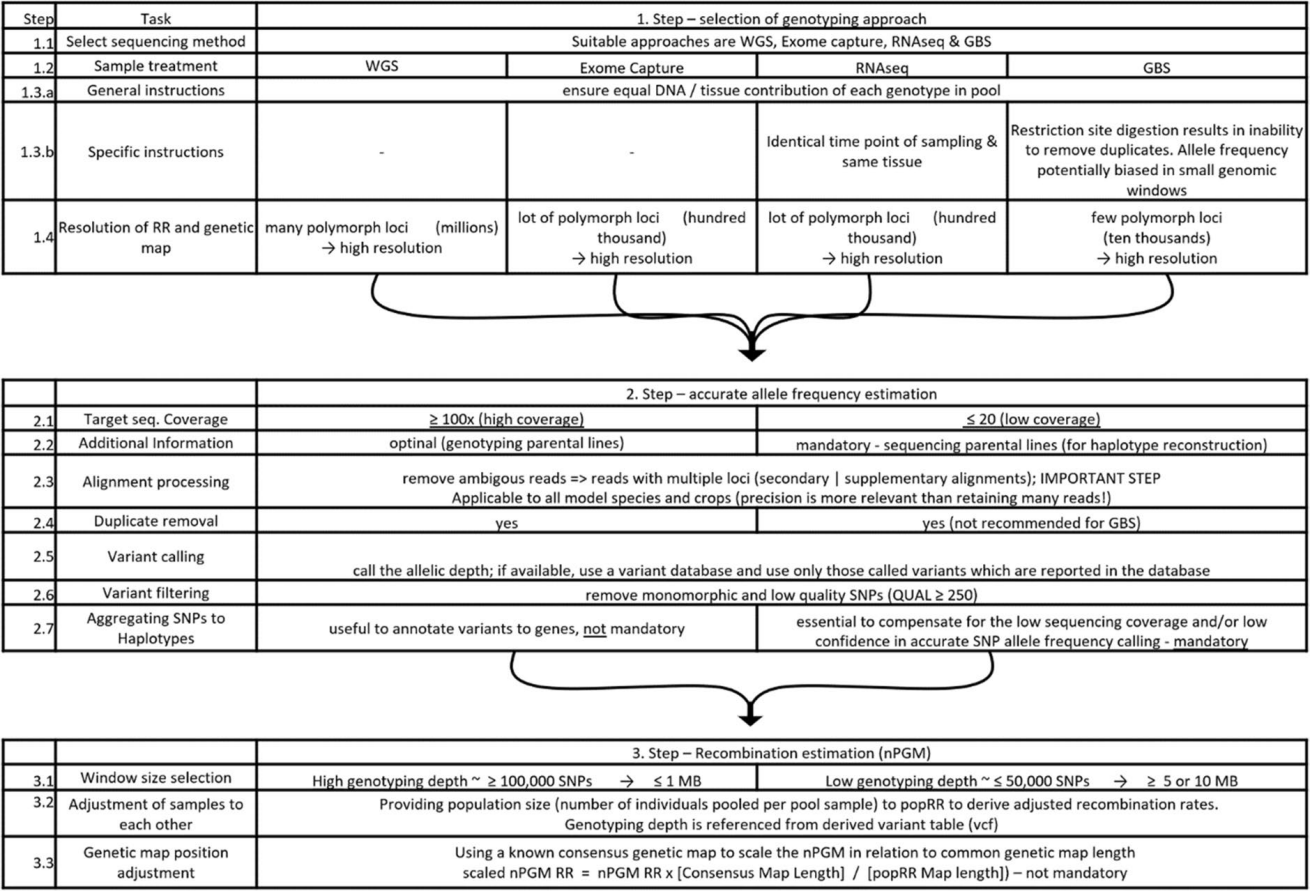
Best practice guideline to estimate the recombination rate from pooled sequencing data

Finally, in case the absolute map length of the PGM is of interest, a genetic map of the variants under consideration is required to scale the observed RR. This step is required, as the presented model cannot accommodate the entire variety of sequencing-induced allele frequency deviations and, thus, was not included in the model fitting. Instead, we propose identifying the typical genetic map extension size in the species of interest and performing a linear scaling of the genetic map position and recombination rate according to Table 2, 3.3.

Beyond the relative RR and map length estimations, this case study presented a method to overcome variations in genotyping depth and the population size by exploiting computer simulations. We recommend the map length adjustment by genotyping depth and population size only in cases where the populations to compare are characterized by highly different numbers of genotypes, the polymorphism count is highly variant, or sequencing depth varies.

## Conclusion

This case study presents a method that allows a cost-efficient estimation of genetic maps and the recombination rate in genomic windows. Our approach exploits the allele frequency and the physical position information. Furthermore, based on computer simulations and experimental data, we have shown that the proposed method allows an accurate assessment of RR. Finally, we have explained how to apply the procedure for other species and discussed potential pitfalls. The functions presented in this publication can be obtained from GitHub *https://github.com/mischn-dev/popRR.git* for both *R* and *Julia* environments, using a filtered VCF file as input.

## Methods

### Consensus map-based population simulations

Our simulations were based on the consensus genetic map generated by Casale et al. (2021). In brief, 45 recombinant inbred line populations have been created by crossing 23 parental inbreds in a double round-robin design [52]. Each of the 4182 RIL was genotyped using a 50 K SNP array [23], and the 45 genetic maps have been integrated. The resulting consensus map comprises 42,077 SNPs with a genetic and physical position [18] (Fig. 1, steps 1–3).

For the simulations, two virtual parental genotypes with different alleles for each of the 42,077 loci were generated and alphaSim [53] was used to derive F2 populations (F1 by crossing, F2 by selfing) with various populations sizes (50; 100; 500; 1000; 2000; 4000; 10,000 genotypes), and various genotyping depths (500; 1000; 2000; 5000; 10,000; 15,000; 20,000; 30,000; 42,077) across the entire genome. These simulations were repeated 20 times for each SNP–genotype count combination (i.e. in total 1260 populations, Fig. 1 – Step 4). For each replicate, a different set of SNPs (except the 42,077 sample) was sampled.

### Recalculation of genetic maps from simulated populations based on Haldane’s mapping function

To estimate the error introduced by the simulation process to the consensus map, we recalculated the Haldane genetic maps for all 20 replicates in populations with 50, 500, and 2000 genotypes. For computational reasons, we ordered the SNPs first by their physical position to realize a correct starting point. Subsequently, the Haldane genetic map (HGM) was calculated using the *qtl* package based on Haldane’s mapping function at an error probability of 0.0001 [54]. Finally, the RR was calculated as the median centiMorgan per megabase pair [cM/MB] value in 50 Mb windows across all variants in this window (Fig. 1, step 6.1).

### Genetic maps from pooled samples

The alleles in a segregating population derived from two parental inbreds are expected to have a frequency of 0.5. However, due to selection or random sampling, the allele frequency at a locus can deviate from this expected frequency. Notably, the deviating allele frequency is expected to attenuate distally toward the expected frequency due to increasing crossover events between the locus and gradually more distal loci [55]. Therefore, the allele frequency and its rate of change should be related to the genetic distance. The genetic map can be generated with as little as one library preparation since genome-wide allele frequency can be determined using whole-genome genotyping or sequencing of a pool of individuals from the population of interest [53]. Our study evaluates whether allele frequency differences across the genome can be used to estimate RRs and genetic maps, even in situations without substantial fitness differences.

We dismissed any individual genotype information after calculating the allele frequency at each SNP across all genotypes by pooling individuals’ genotypic information (Fig. 1, step 5.1). We estimated the factor *K*_*M1M2*_ as:

1 $${K}_{M1M2}=\frac{\Delta {AF}_{M1M2}}{{\mathit{\log}}_{10}\Delta {Dist}_{M1M2}}$$

where Δ*AF*_*M1M2*_ was the allele frequency deviation of the considered physically neighboring SNP pair (M1, M2) and log_10_ *Δdist* the decadic logarithm of the physical distance between them. The factor *K*_*M1M2*_, which comprises the two SNPs’ relative recombination rate, was added up along the chromosome to generate a pool genetic map (PGM, Fig. 1, step 6.2). As the absolute size of factor *K*_*M1M2*_ can not be interpreted, it needs to be scaled first. In the first step, we adjusted the PGM using the *adj*_*start*_ correction factor, which was calculated as the ratio between the length of the consensus map across all chromosomes (ML_ref_) in cM and the sum of the PGM across all chromosomes (ML_PGM_). An adjustment value *adj*_*start*_ was calculated separately for each simulated sample (Fig. 1, steps 7 & 8).

The above-described correction factor *adj*_*start*_ was used to estimate the effect of the genotyping depth (*Markers*) and population size (*Genotypes*) on the map length in order to realize in the next step a correction of the map length for these two factors. Therefore, we evaluated a simple linear model without intercept (Eq. 2; Fig. 1, step 8.1):

2 $${adj}_{start}=a\ast \sum Markers+b\ast \sum Genotypes$$

and a non-linear least square model (nls, Eq. 3; Fig. 1, step 8.2):

3 $${adj}_{start}=\alpha \ast {e}^{\beta \ast {\mathit{\log}}_2\sum Markers}\ast {e}^{\gamma \ast {\mathit{\log}}_2\sum Genotypes}+\frac{\theta }{\sqrt{\sum Markers}}$$

and compared them concerning AIC and log-likelihood to identify the best fitting model.

The nls model described above comprised four sub transformations (α, β, ϒ, Θ) and the SNP and genotype count were log_2_ transformed. For both models, the parameters were estimated across all simulated 1260 populations. Based on these estimates, the correction factor adj_start_ was calculated using each population’s genotyping depth and population size (Fig. 1, step 8.3).

According to the observed log-likelihood and AIC, the nls model was used in all following analyses and multiplied to each SNP’s *K* value to generate a corrected PGM estimate (nPGM), (Eq. 4; Fig. 1, step 9).

4 $${K}^{\prime }={K}_{M1M2}\ast {adj}_{start\ (nls)}$$

### RR estimation from adjusted pool genetic map (nPGM)

RR [cM/MB] was calculated from the nPGM for each SNP pair. Next, an average RR value was calculated for 50 Mb windows, applying a sliding window approach (window size 50 MB, slide 0.5 x window size).

Finally, the RR of the simulated populations with 50, 500, and 2000 genotypes on all genotyping depths was compared between (i) nPGM (RR_nPGM_) and the consensus map (RR_consensus_) (ii) HGM (RR_HGM_) and the consensus map (Fig. 1, step 10), and (iii) nPGM (RR_nPGM_) and HGM (RR_HGM_).

### nPGM calculation in experimental populations

The previously described 45 HvDRR populations were used to estimate the nPGM in experimental populations and compare the RR_nPGM_ to the RR_HGM_. The HGM was calculated as described by Casale et al. (2021). For the nPGM construction of each population, monomorphic SNPs and SNPs with identical or missing physical positions were omitted. In addition, SNPs with more than 10% missing information were omitted as well. Finally, the allele frequency was calculated, and the nPGM was derived from it, as was described above. The nPGM was used to estimate the RR_nPGM_ (Fig. 1, steps II & II.i). The RR_nPGM_ estimate accuracy was assessed by comparing it to RR_HGM_.

### Impact of sequencing error on the pool genetic map estimation accuracy

In the above-explained simulations, the allele frequency was calculated from the genotypic information of individual samples. However, the primary purpose of our nPGM approach was the recombination estimation from pool sequencing data. Therefore, based on the allele frequency of the individual genotyping simulations, we performed a pool sequencing simulation to estimate the effect of both the sequencing and sampling error on the genetic map estimation accuracy using the nPGM approach (Fig. 1, step 11). Therefore, we selected four scenarios, characterized by a genotyping depth of 10,000 and 42,077 markers and a population size of 50 and 100 genotypes.

The *simReads* function of the Rsubread package [56] was used to simulate the sequencing data based on the allele frequency of the simulated populations and the barley reference genome (Barley Morex V2 pseudomolecules [57]; Fig. 1, step 5.2). s*imReads* created a fastq file with a locus coverage of approximately 3000 reads per locus. From this set, three sequencing depths were sampled (10, 50 & 100 reads per locus) with ten replicates per combination of either 10,000 or 42,077 variants and 50 or 100 genotypes (*Sequencing depth x Genotyping depth x population size*).

In the next step, the subsets of simulated 100 bp long paired-end reads were aligned to the Barley Morex V2 pseudomolecules reference genome by *bwa mem* [58]. Following, the reads were filtered by omitting all reads with an alignment score below 60 using *samtools* [59]. Next, the variants were called from the aligned reads using *samtools 1.8 mpileup* and *bcftools 1.8 call* [60], where all reads with a variant quality below 30 were omitted.

Finally, the allele frequency and physical positions were extracted and based on eq. 5, a pool sequencing derived nPGM, named nPGM_ps_, was calculated (Fig. 1, step11.1). Next, we estimated from the nPGM_ps_ the RR_nPGMps_ and compared it in 10, 25, and 50 MB windows across the genome to the RR_consensus_ and RR_nPGM_. Furthermore, the two variant levels (10,000, 42,077) were compared to assess the effect of the genotyping depth in the pooling strategy.

### Estimation of general recombination effect of parental inbreds

We calculated the general recombination effect of each of the 23 parental inbreds based on the nPGM, and compared it against the values reported by Casale et al. (2021). We used the same G-BLUP model to retain consistency in comparing both HGM and nPGM approaches. If not mentioned differently, all analyses were performed in R 4.0.2 [61] and Julia 1.6.2 [62].

## Supplementary Information

**Additional file 1: Suppl. Figure 1.** Deviation between observed and expected allele frequency (y-axis) for different numbers of genotypes per population (x-axis). The expected allele frequency value in an F2 population of infinite size is 0.5 and was set as the expected allele frequency. The observed allele frequency results from simulating a population with a given genotype count by AlphaSim. Each dot presents one simulated population. A total of 1260 populations were simulated. **Suppl. Figure 2.** Linear model (magenta) and non-linear least square (turquoise) models to predict the impact of a population’s size and genotyping depth on the map extension (length). The model estimate on the y-axis is based on the pool genetic map estimation. Each point illustrates an individual population. The dashed line indicates the ideal fit. (step 8.1 & 8.2 in Fig. 1). **Suppl. Figure 3.** Marey map of genetic position (y-axis) against the physical position (x-axis) for all 45 experimental populations. The nPGM (coral) is compared against the HGM (blue). Chromosomes and populations are faceted. **Suppl. Figure 4.** The genome-wide general recombination effect for each parental inbred line, computed using a GBLUP model, based on the nPGM genome-wide RR observations. **Suppl. Figure 5.** The correlation plot of the RR from pooled genotyping (y-axis) compared to the RR from simulated pool sequencing (x-axis) at 100 reads coverage in 10 MB (**A**) and 50 MB (**B**) genomic windows. Four samples, differing in marker or genotype count, are indicated by the numbers 1 to 4 for both A and B. The number of variants in the genomic windows is indicated by color, while the chromosomes are differentiated by shape. **Suppl. Figure 6.** The effect of the median marker distance on the RR_nPGM_ to RR_HGM_ correlation coefficients across all HvDRR populations. **A** - the effect of median marker distance (bp) on the Pearson correlation. **B** – the effect of the median marker distance on the Spearman correlation. **C –** the genome-wide distribution of inter-marker distance (bp) for four HvDRR populations, characterized by a low (yellow, HvDRR08, HvDRR43) and a high (turquoise, HvDRR11, HvDRR43) RR_nPGM_ to RR_HGM_ Pearson correlation.

## Data Availability

Code with example data sets and explanations to perform the analysis are provided at https://github.com/mischn-dev/popRR.git*.* HvDRR population information and genetic maps can be found in Casale et al. (2021).

## Abbreviations

GRE: General recombination estimate
HGM: Haldane genetic map
HvDRR: Spring barley double round-robin populations
Lm: Linear model
MB: Megabase pairs
Nls: Non-linear least square model
nPGM: Non-linear adjusted pool genetic map
nPGM_ps_: Non-linear adjusted pool genetic map derived from pool sequencing
ML: Map length
PGM: Pool genetic map
RIL: Recombinant inbred line
RR: Recombination rate
SNP: Single nucleotide polymorphism
