# Supplementary material for: Accurate recombination estimation from pooled genotyping and sequencing: a case study on barley
Source: BMC Genomics. 2022 Jun 25;23:468. doi: 10.1186/s12864-022-08701-7 (PMC9233355; doi:10.1186/s12864-022-08701-7)
Supplement: Supplementary file 1 — Additional file 1: Suppl. Figure 1. Deviation between observed and expected allele frequency (y-axis) for different numbers of genotypes per population (x-axis). The expected allele frequency value in an F2 population of infinite size is 0.5 and was set as the expected allele frequency. The observed allele frequency results from simulating a population with a given genotype count by AlphaSim. Each dot presents one simulated population. A total of 1260 populations were simulated. Suppl. Figure 2. Linear model (magenta) and non-linear least square (turquoise) models to predict the impact of a population’s size and genotyping depth on the map extension (length). The model estimate on the y-axis is based on the pool genetic map estimation. Each point illustrates an individual population. The dashed line indicates the ideal fit. (step 8.1 & 8.2 in Fig. 1). Suppl. Figure 3. Marey map of genetic position (y-axis) against the physical position (x-axis) for all 45 experimental populations. The nPGM (coral) is compared against the HGM (blue). Chromosomes and populations are faceted. Suppl. Figure 4. The genome-wide general recombination effect for each parental inbred line, computed using a GBLUP model, based on the nPGM genome-wide RR observations. Suppl. Figure 5. The correlation plot of the RR from pooled genotyping (y-axis) compared to the RR from simulated pool sequencing (x-axis) at 100 reads coverage in 10 MB (A) and 50 MB (B) genomic windows. Four samples, differing in marker or genotype count, are indicated by the numbers 1 to 4 for both A and B. The number of variants in the genomic windows is indicated by color, while the chromosomes are differentiated by shape. Suppl. Figure 6. The effect of the median marker distance on the RRnPGM to RRHGM correlation coefficients across all HvDRR populations. A - the effect of median marker distance (bp) on the Pearson correlation. B – the effect of the median marker distance on the Spearman correlation. C – the genome-wide dist [file 12864_2022_8701_MOESM1_ESM.docx]

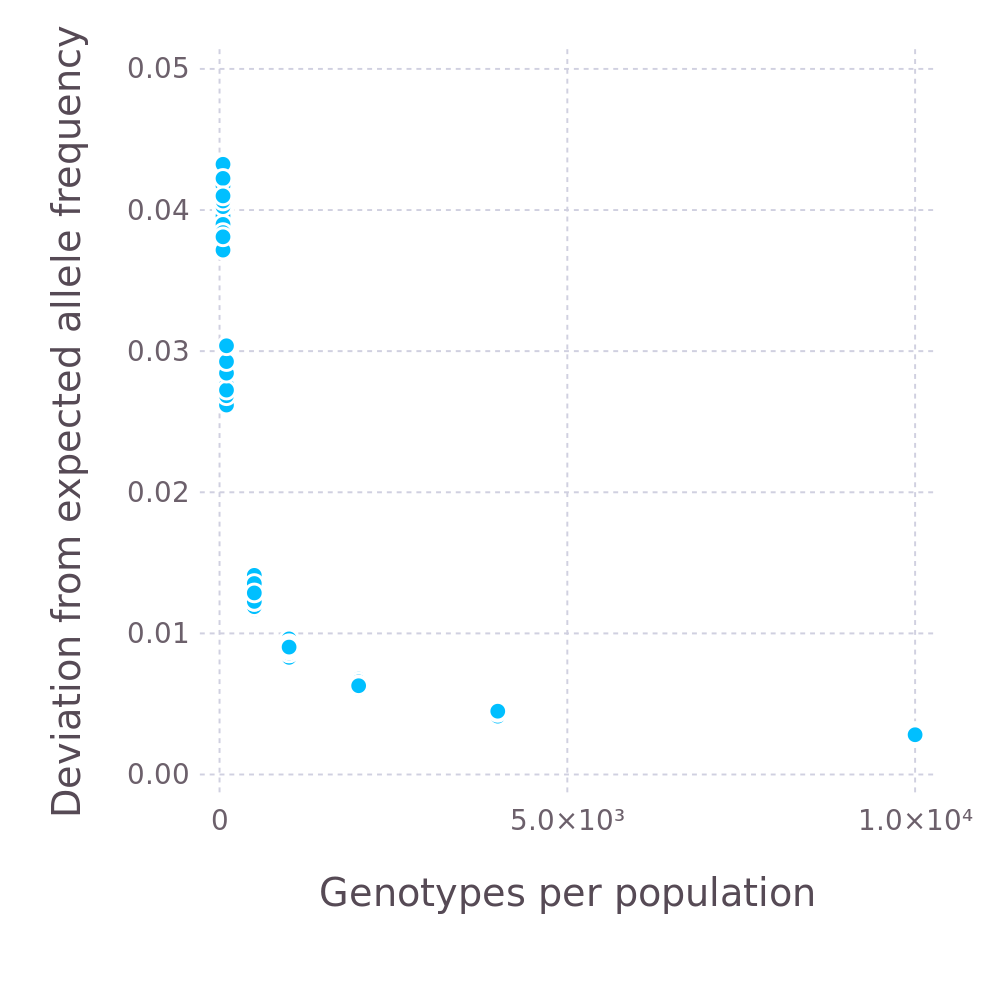
**Suppl. Figure 1:** Deviation between observed and expected allele frequency (y-axis) for different numbers of genotypes per population (x-axis). The expected allele frequency value in an F2 population of infinite size is 0.5 and was set as the expected allele frequency. The observed allele frequency results from simulating a population with a given genotype count by AlphaSim. Each dot presents one simulated population. A total of 1260 populations were simulated.


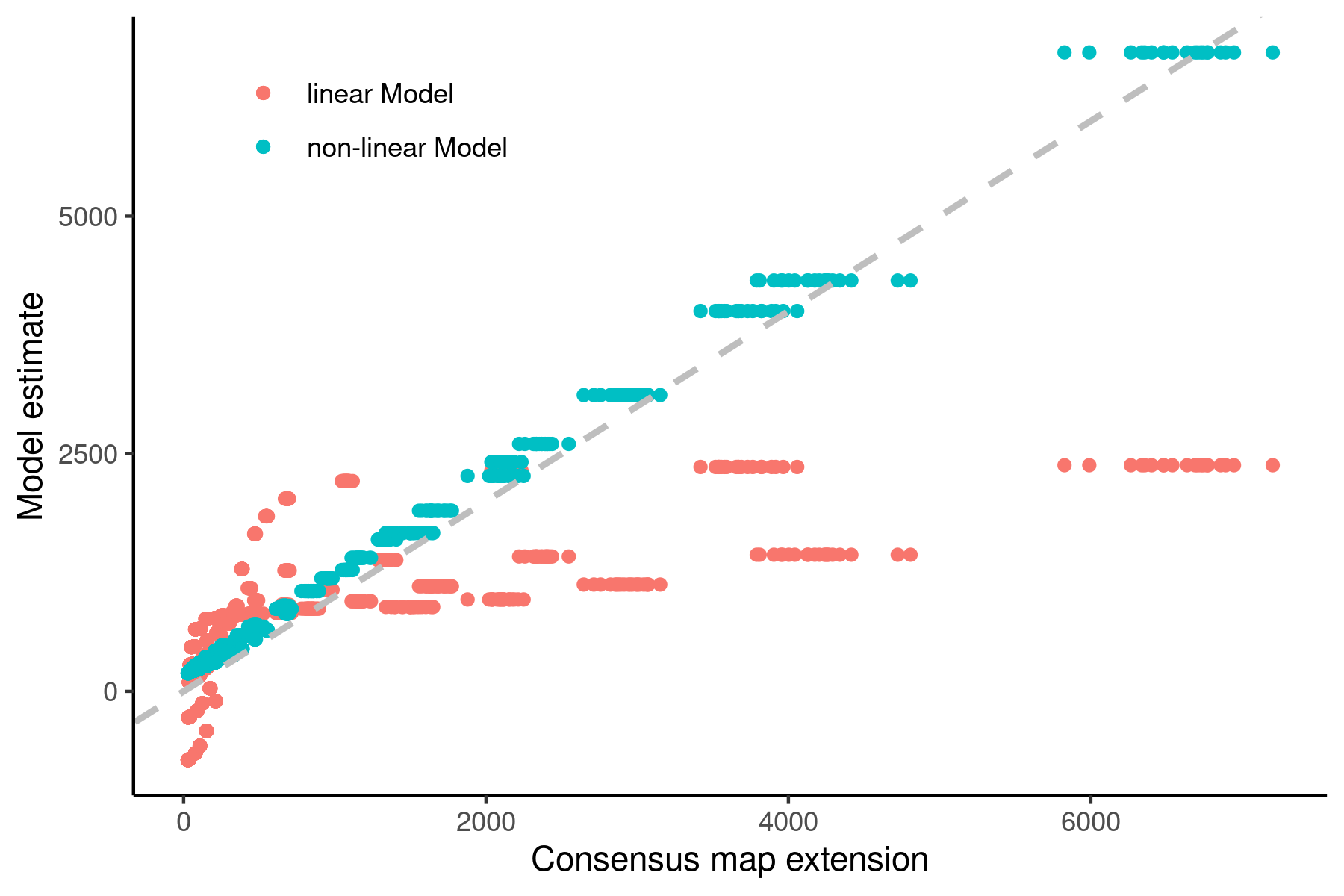


**Suppl. Figure 2:** Linear model (magenta) and non-linear least square (turquoise) models to predict the impact of a population‘s size and genotyping depth on the map extension (length). The model estimate on the y-axis is based on the pool genetic map estimation. Each point illustrates an individual population. The dashed line indicates the ideal fit. (step 8.1 & 8.2 in figure 1)


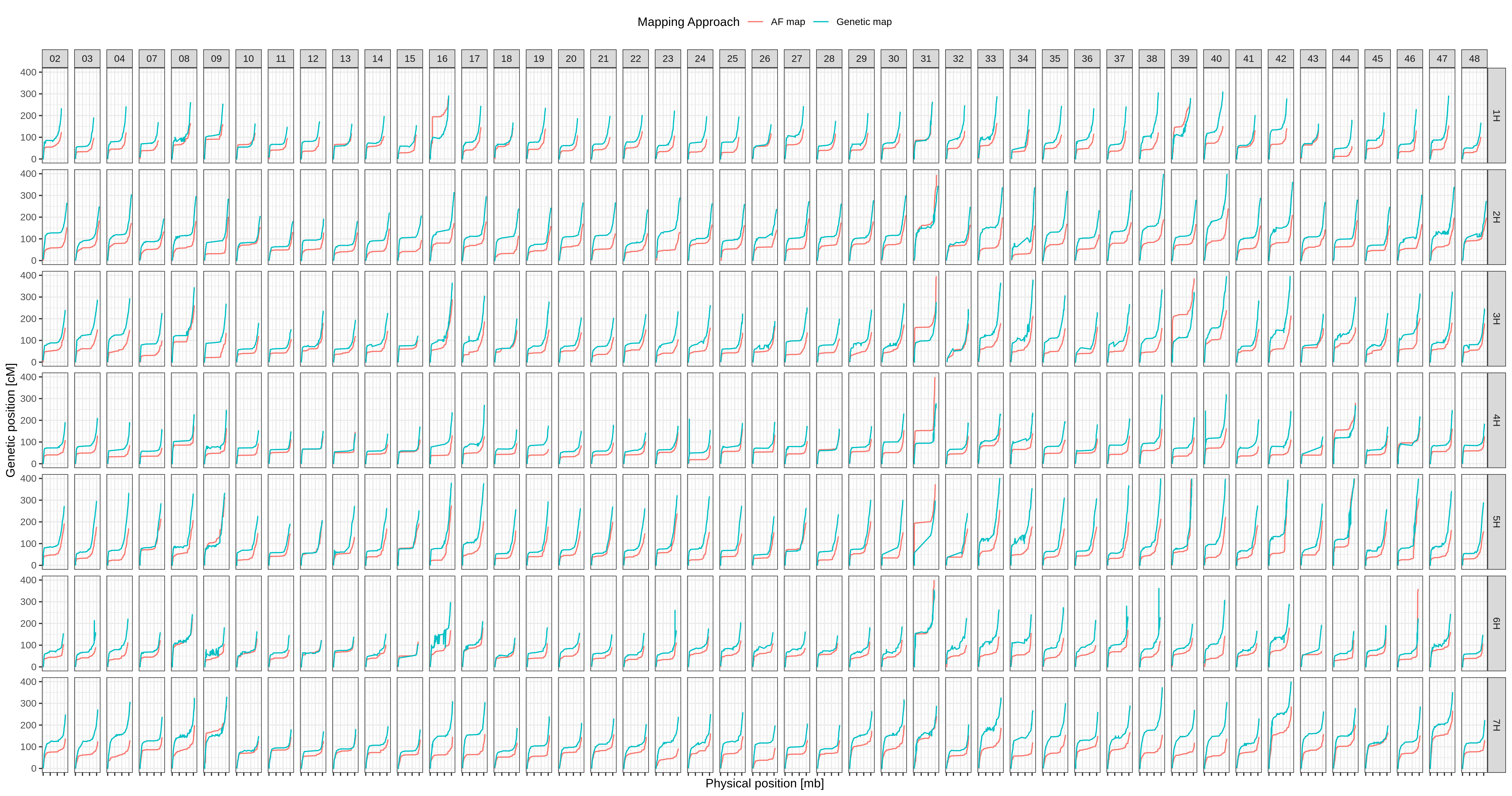


**Suppl. Figure 3:** Marey map of genetic position (y-axis) against the physical position (x-axis) for all 45 experimental populations. The nPGM (coral) is compared against the HGM (blue). Chromosomes and populations are faceted.


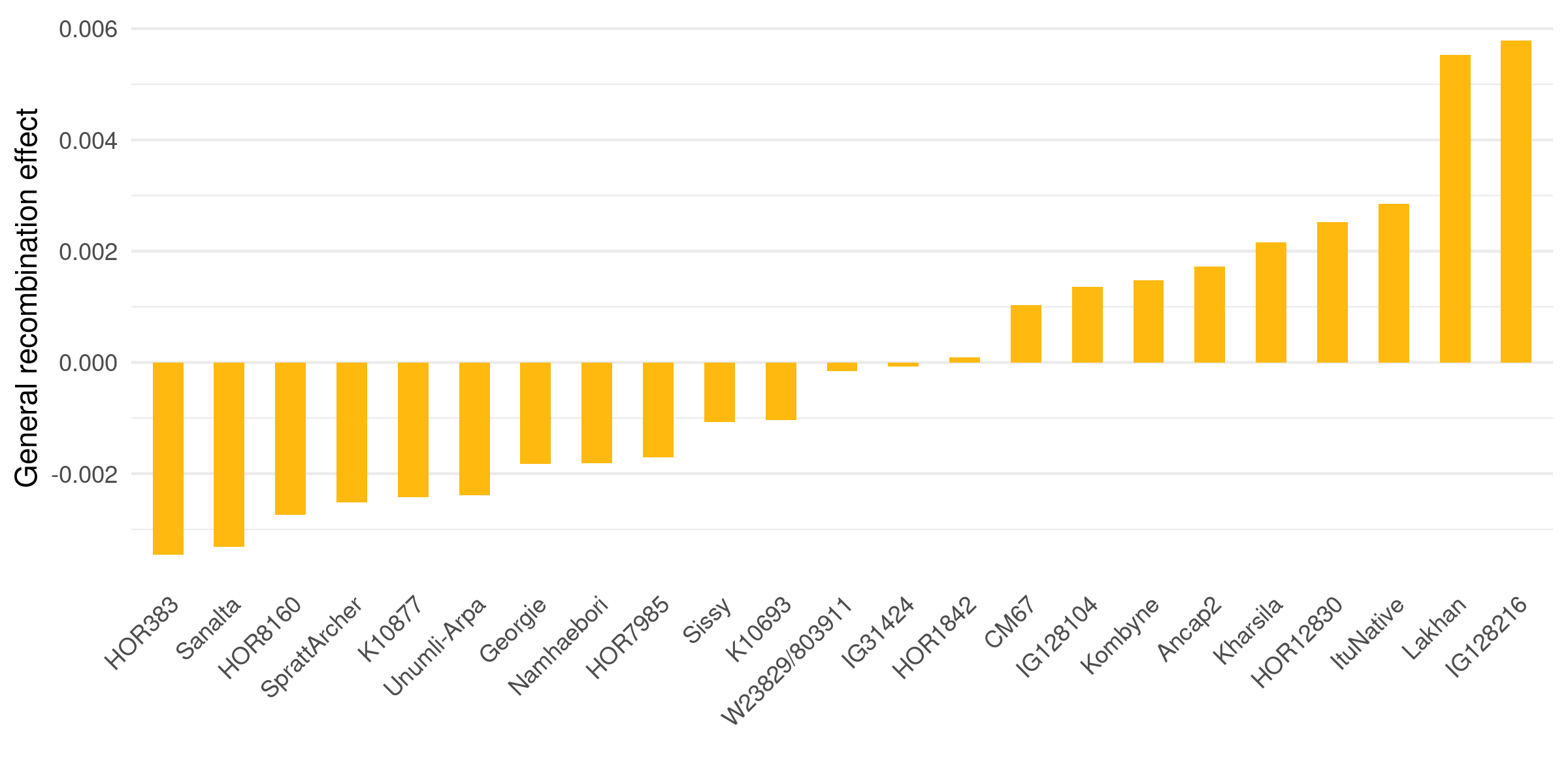


**Suppl. Figure 4:** The genome-wide general recombination effect for each parental inbred line, computed using a GBLUP model, based on the nPGM genome-wide RR observations.


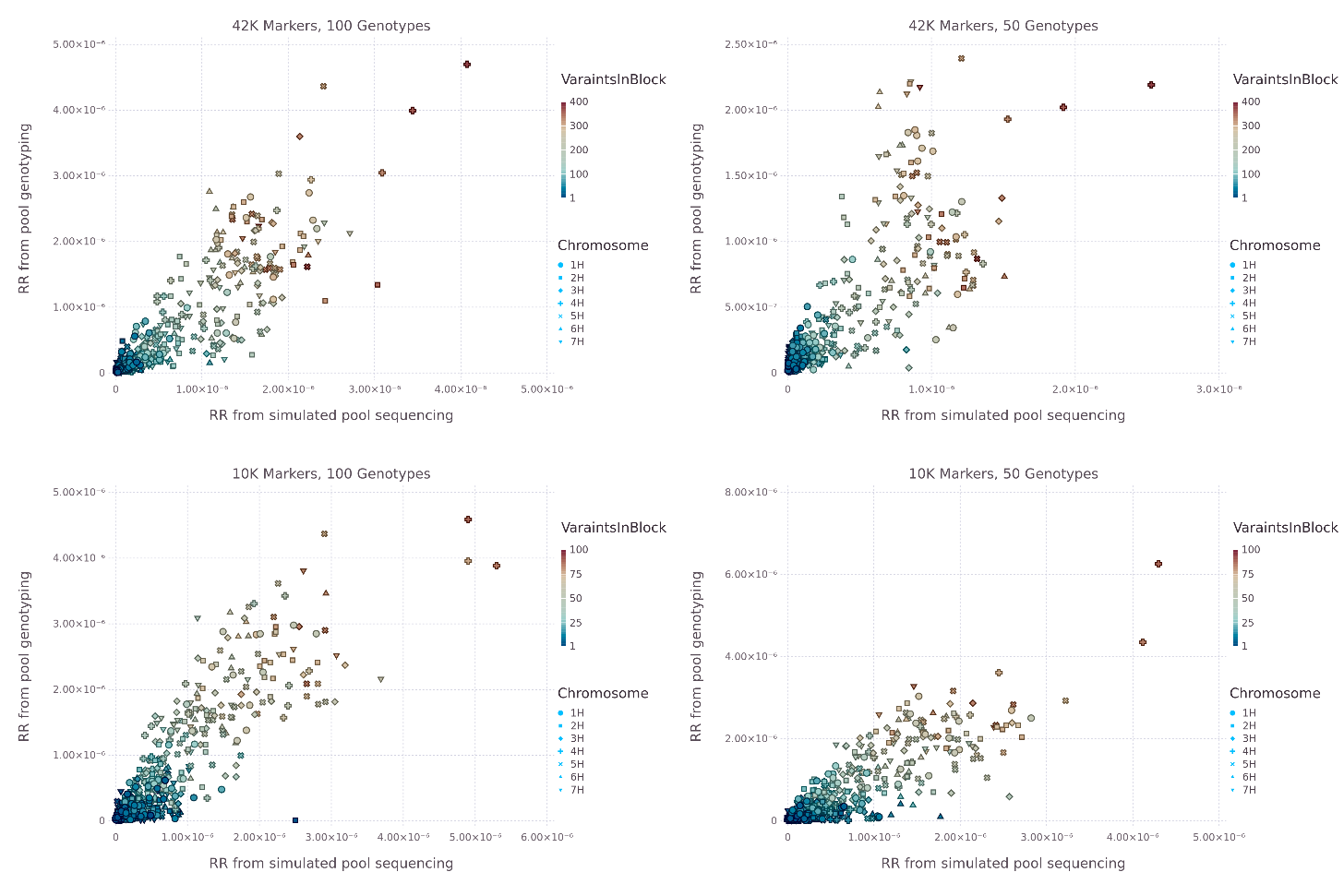


A

A1

A2

A3

A4

**
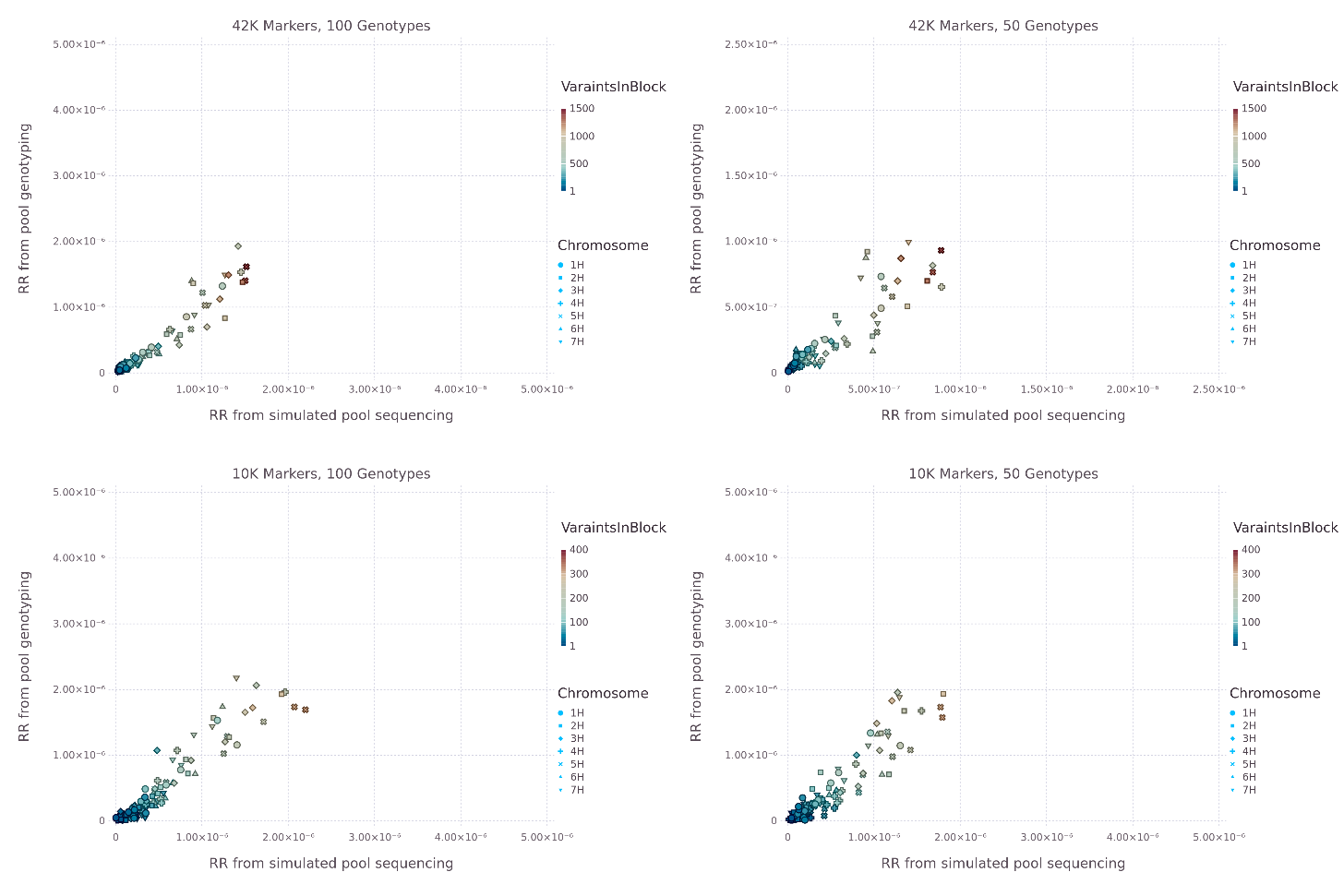
Suppl. Figure 5:** The correlation plot of the RR from pooled genotyping (y-axis) compared to the RR from simulated pool sequencing (x-axis) at 100 reads coverage in 10 MB (**A**) and 50 MB (**B**) genomic windows. Four samples, differing in marker or genotype count, are indicated by the numbers 1 to 4 for both A and B. The number of variants in the genomic windows is indicated by color, while the chromosomes are differentiated by shape.

B

B1

B2

B4

B3


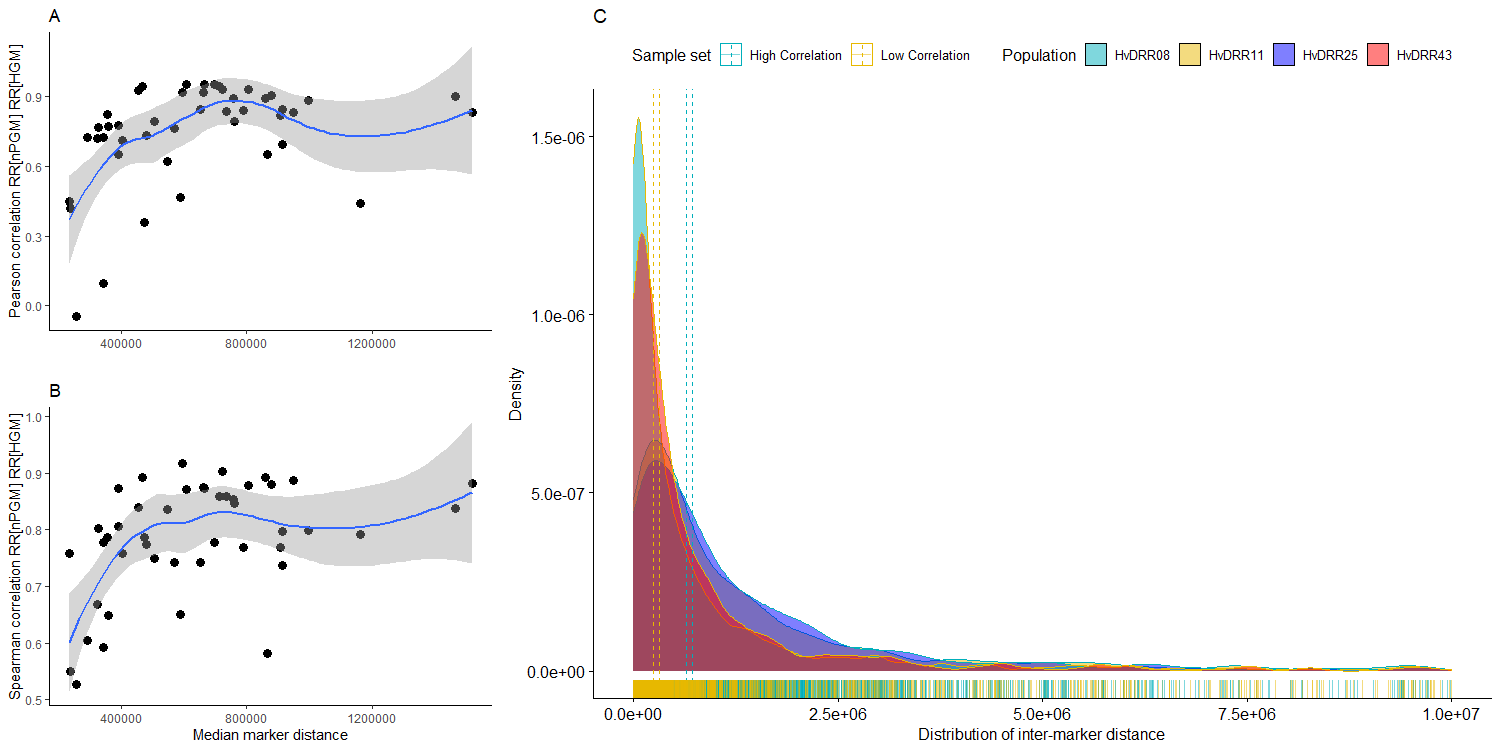


**Suppl. Figure 6**: The effect of the median marker distance on the RR_nPGM_ to RR_HGM_ correlation coefficients across all HvDRR populations. **A** - the effect of median marker distance (bp) on the Pearson correlation. **B** – the effect of the median marker distance on the Spearman correlation. **C –** the genome-wide distribution of inter-marker distance (bp) for four HvDRR populations, characterized by a low (yellow, HvDRR08, HvDRR43) and a high (turquoise, HvDRR11, HvDRR43) RR_nPGM_ to RR_HGM_ Pearson correlation.
